# Scaling and responses of extreme hourly precipitation in three climate experiments with a convection-permitting model

**DOI:** 10.1098/rsta.2019.0544

**Published:** 2021-04-19

**Authors:** Geert Lenderink, Hylke de Vries, Hayley J. Fowler, Renaud Barbero, Bert van Ulft, Erik van Meijgaard

**Affiliations:** ^1^ KNMI, RDWK, De Bilt, The Netherlands; ^2^ Geoscience & Remote Sensing, Delft University of Technology, Delft, The Netherlands; ^3^ School of Engineering, Newcastle University, Newcastle-upon-Tyne, UK; ^4^ INRAE, RECOVER, Aix-en-Provence, France

**Keywords:** hourly precipitation extremes, climate change, precipitation scaling

## Abstract

It is widely recognized that future rainfall extremes will intensify. This expectation is tied to the Clausius-Clapeyron (CC) relation, stating that the maximum water vapour content in the atmosphere increases by 6–7% per degree warming. Scaling rates for the dependency of hourly precipitation extremes on near-surface (dew point) temperature derived from day-to-day variability have been found to exceed this relation (super-CC). However, both the applicability of this approach in a long-term climate change context, and the physical realism of super-CC rates have been questioned. Here, we analyse three different climate change experiments with a convection-permitting model over Western Europe: simple uniform-warming, 11-year pseudo-global warming and 11-year global climate model driven. The uniform-warming experiment results in consistent increases to the intensity of hourly rainfall extremes of approximately 11% per degree for moderate to high extremes. The other two, more realistic, experiments show smaller increases—usually at or below the CC rate—for moderate extremes, mostly resulting from significant decreases to rainfall occurrence. However, changes to the most extreme events are broadly consistent with 1.5–2 times the CC rate (10–14% per degree), as predicted from the present-day scaling rate for the highest percentiles. This result has important implications for climate adaptation.

This article is part of a discussion meeting issue ‘Intensification of short-duration rainfall extremes and implications for flash flood risks’.

## 1. Introduction

Sub-daily precipitation extremes can cause local flash floods, strong soil erosion and landslides, and are therefore a potential threat to society. Because these extremes are mostly related to summertime convective storms, with complex dynamics at cloud scales up to the atmospheric mesoscale of several hundreds of km, they are difficult to predict not only in numerical weather predictions, but also in a climate change context due to a lack of resolution in climate models and/or relative short simulations. Understanding these convective cloud dynamics and the related atmospheric processes is still limited, particularly in a climate change context [1,2].

Despite the limited understanding of many local processes, the increase in atmospheric moisture provides a rather robust estimate of the order of magnitude change to precipitation extremes. Taking dew point temperature (or temperature assuming unchanged relative humidity), each degree of warming is equivalent to 6–7% more water vapour according to the well-known Clausius-Clapeyron (CC) relation. The CC rate is widely considered as the typical benchmark to estimate changes in precipitation extremes in a warming climate [3].

Surprisingly, observationally based relations sometimes appear to deviate substantially from the CC rate, in particular for short-duration precipitation extremes. As an example, 10 min rainfall extremes derived from Dutch observations show a very consistent dependency of 13% increase per degree over almost a 20-degree dew point temperature range [4]. Besides this dependency being close to two times the CC rate, the apparently simple, regular behaviour is also remarkable, given that summer rain storms are very intermittent, chaotic and influenced by multiple atmospheric drivers. For hourly precipitation extremes, a consistent dependency of close to, or slightly below, 2CC has been found from pairing precipitation data with near-surface dew point temperature [5,6]. Precipitation data from Hong Kong and the Netherlands also show similar scaling rates when paired with surface dew point temperature [7], suggesting that super-CC scaling may apply to broader regions and different climates. While not reaching 2CC, scaling rates exceeding CC have been found in many areas [8–10].

The approach whereby precipitation is paired with (surface) temperature or dew point temperature observations, and dependencies of extremes on (dew point) temperature are derived will be termed ‘apparent’ scaling. We used apparent scaling here to comply with the terminology used in previous papers [11–13] and with ‘apparent’ we do not refer to the usefulness of the approach. The value of apparent scaling rates in a climate change context has been criticized in a number of recent studies [11,14–17]. Additionally, the causes and interpretation of the observed apparent scaling rates, in particular those exceeding the CC rate, have also been heavily debated.

A long-standing hypothesis is that super-CC scaling is caused by a purely statistical effect, whereby rainfall at lower temperatures is dominated by large-scale rain systems, with moderate intensities, whereas intense convective systems with intense rainfall dominate at higher temperatures [18]. This could give rise to a steeper dependency on (dew point) temperature, exceeding the CC rate, even when both rain types behave according to the CC rate. While some studies have provided evidence for this theory [19–21], other studies have found evidence for super-CC behaviour of convective extremes [4,6,9,22,23]. Likewise, changes in atmospheric conditions tied to the seasonal cycle have been proposed to influence scaling rates [14]. Finally, it has been proposed that super-CC scaling may be due to the use of percentiles conditional on the occurrence of rain [16,24]. Given these potential caveats, one may question the relevance of apparent scaling in understanding future changes to precipitation extremes. Sensitivities to short-term variability, called apparent scaling, may not be representative of long-term changes to precipitation extremes due to climate change, hereafter called climate scaling.

We believe that a considerable part of the criticism on the usefulness of apparent scaling is related to the fact that most papers on scaling use dry bulb temperature as the scaling variable [12,13,25,26]. The majority of published scaling studies use dry bulb temperature—often because humidity data is lacking—and with dry bulb temperature widely varying scaling behaviour has been obtained, highly dependent on location and season. The fundamental problem with (dry bulb) temperature scaling—as opposed to dew point temperature scaling—is that temperature alone is not a reliable indicator of the actual humidity of the air. Therefore, if higher temperatures are not associated with higher moisture content (roughly following the CC rate), it is hard to see what one could learn from *temperature* scaling in a climate change context; indeed it is the actual moisture increase of the atmosphere which is responsible for the expected increase in precipitation extremes, not the temperature itself [3].

In this paper, we will closely examine the climate scaling of hourly extremes derived from comparing future periods with the present day, in relation to apparent scaling derived from present-day climate variability, in three sets of simulations with a convection-permitting regional climate model (CPRCM). In the first set—consisting of only 10 summer months—present-day weather is repeated under a uniform or surrogate warming (SW) scenario assuming unchanged relative humidity [27]. In the second set, consisting of 11 years of simulation, a more elaborate pseudo-global warming (PGW) approach is used, by applying changes in temperature, humidity and winds that vary in space and per month [28–30]. In the third set, also consisting of 11 years of simulation, boundary conditions are taken from a Global Climate Model (GCM). At a conceptual level, these sets take various aspects of the climate response into account. The SW set only looks at the thermodynamic effect, with a relatively small stability change only related to the convective activity itself [31]. The PGW approach samples substantial changes in relative humidity and stability and includes systematic changes to the large-scale circulation—changes in pressure related to geostrophically balanced flow associated with the density temperature/humidity gradients [29]. Finally, the GCM-driven simulations include changes to all atmospheric drivers, including changes in day-to-day synoptic variability.

With these simulations, we focus on the following questions:

1. Can we find evidence of climate scaling rates exceeding the CC rate as suggested by apparent scaling? Does this depend on the experimental setup with different degrees of thermodynamic and dynamical changes? Does this depend on the return level or percentile of the extreme?
2. Can we use the scaling diagram to relate the climate change response to characteristics of the apparent scaling? And alternatively, can we use the apparent scaling framework differently to understand for which dew point temperature ranges precipitation extremes are most sensitive to warming?

## 2. Methods

### (a) Model and experiment

The simulations are performed with the non-hydrostatic CPRCM HCLIM-AROME (cycle 38) [32]. The parameterization of deep cumulus convection is completely turned off, whereas shallow convection is still parameterized. We analysed the total sum of the rainfall, including a small contribution from the shallow convection scheme.

**Figure 1.**
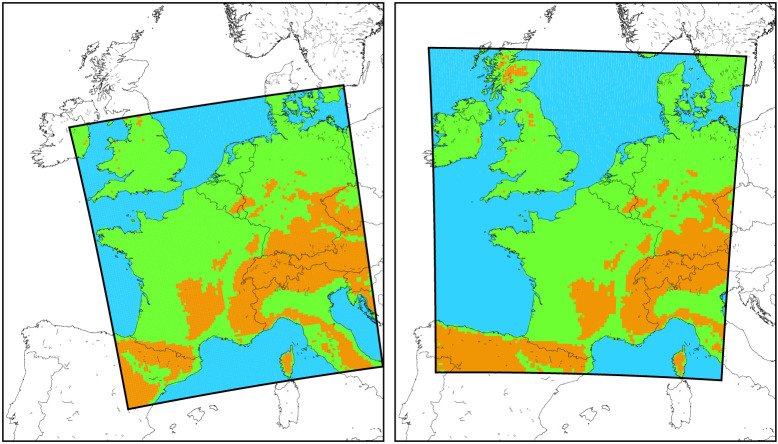
Analysis domains (simulation domain with 100 grid points cut off at the boundaries) for the experiments. Low-altitude land areas with an average height of the 5 × 5 grid boxes below 400 m are green, and areas above that height are brown. (Online version in colour.)

Experiments are performed for two domains, both at 2.5 km resolution and for approximately 900 × 900 grid points: one centred over Central Western Europe (CXFPS) and one shifted slightly westward (EUCP_NW). The latter domain has been configured according to contractual obligations in the European EUCP project (https://www.eucp-project.eu/) and overlaps by 90% with the CXFPS domain (figure 1). For practical purposes, we do not distinguish between the two domains. All experiments use simulations at 12 km resolution with the regional climate model RACMO as an intermediate step between the driving GCM/global reanalysis data and the CPRCM, the latter receiving boundary conditions at hourly time intervals.

Three sets of experiments have been performed to study the climate change response of hourly extremes:

1. E_SW_: A set of 10 individual summer months from the period after the year 2000 for present-day and perturbed to warmer conditions. This is an existing experiment, which was targeted at understanding the influence of humidity in a very simplified experiment setting, and wet months were selected to produce a relatively large sample of rainfall events in relatively short simulation [31]. Runs for present-day climate are performed using ERA-Interim as boundary conditions. For the future runs, the boundary conditions entering RACMO are perturbed by a 2 degree uniform warming in the horizontal as well as the vertical (in pressure) and constant relative humidity (and adjusting the absolute humidity accordingly). By design, this experiment only captures the thermodynamic (moisture-driven) response to warming, including local dynamical feedbacks. But, this experiment also includes a small stabilization of the atmosphere due to the temperature perturbation imposed at different pressure levels and an adjustment due to the convective processes which tends to lead to an adjustment towards a moist adiabatic lapse rate [31].
2. E_PGW_: A pseudo-global warming (PGW) experiment, where we impose the full three-dimensional change structure in temperature, humidity, winds and pressure from a climate change experiment. In this case, we used the mean change per month following Prein *et al*. [30], but here derived from a 16-member ensemble with the ECEARTH global climate model [33]. Changes are derived by comparing 2052–2061 with 2009–2018, corresponding to a global temperature rise of 1.5 degrees. The control period is 2008–2018, and again ERA-Interim boundary conditions are used. Perturbations in the PGW experiment are merged with the boundary conditions driving RACMO; greenhouse gas concentrations and aerosol background concentrations are taken from the 10-year period corresponding to 1.5 K temperature rise in ECEARTH. The PGW experiment captures changes in relative humidity, vertical temperature stratification, as well as systematic changes in the large-scale circulation and pressure, including the drying response resulting from anomalous high pressure over the eastern Atlantic and Great Britain.
3. E_GCM_: A GCM-driven simulation for both the control period, 1995–2005, and the future period, 2089–2099, driven by ECEARTH boundary conditions. We used particular members of the ECEARTH 16-member initial-conditions ensemble, which were chosen to be middle-of-the-road in a number of circulation and precipitation statistics; that is, they behave close to the ensemble mean (see electronic supplementary material). This experiment contains the full response to thermo-dynamical as well as dynamical processes, including changes to day-to-day synoptic scale variability, and is considered the most realistic in this respect. The downside of this experiment is that it is too dry and too cold, by approximately 1–2 degrees in temperature as well as dew point temperature, due to a bias in the global climate model ECEARTH [34].

Finally, table 1 lists the available runs, and figure 1 shows the domains used in this study.

**Table 1.** Overview of the available runs with HCLIM. The ERA-interim driven model integration 1999–2009 on the CXFPS domain is only used for evaluation purposes. Note that we analysed the full 11-year period of the simulations in order to improve the statistics, including the first year of the simulation which is usually used as a spin up of the model. We think this is acceptable considering that we only analyse the period from May to October.

experiment	domain	ERA-interim	perturbation	GCM control	GCM future
E_SW_	CXFPS	10 selected summer months	+2 degree and constant RH	—	—
E_PGW_	EUCP_NW	2008–2018	full PGW derived at 1.5 degree global warming	—	—
E_GCM_	CXFPS	1999–2009	—	ECEARTH	ECEARTH
				member 14	member 4
				1995–2005	2089–2099

### (b) Statistical analysis

In this paper, we have analysed grid point scale (2.5 × 2.5 km^2^) hourly precipitation. In most of the paper, we use a coarse-graining procedure by considering two statistics from (non-overlapping) blocks of 5 × 5 grid points: pr_max_, the maximum value of the 25 grid points per block for each hourly interval, and pr_sample_, a sample value taken from the centre point of the 5 × 5 block. This procedure is the same as in Lenderink *et al*. [31] and keeps the data volume relatively low and removes some of the spatial dependencies. Since we are interested in extremes we mostly use pr_max_, because this guarantees that we capture all of the extreme values in the limited dataset. We only used data for the six months, May to October, in order to capture the extremes in the warm season, realizing that hourly extremes also occur in late spring and early to mid-autumn [35,36].

For apparent scaling, we used a binning approach pairing hourly precipitation to hourly dew point temperature taken 4 h earlier giving the most robust results in the earlier analysis [7]. Bins are 2 degrees wide and overlap by one degree. Percentiles are computed using only the wet events, above a 0.1 mm threshold. Scaling curves are computed from data pooled over large areas, up to the full analysis domain, for both the control as well as the future simulation. To interpolate the scaling curves, a LOESS filter is used (with a small filter width of 0.6). Practically, this is very close to linear interpolation, with only small smoothing effects.

The climate change scaling rates—comparing the future simulation with the present-day simulation—in hourly extremes of pr_max_ is also computed from data pooled over large areas. We used only land points, omitting the area within 250 km from the boundaries and points over the sea. Note that the area cut-off at the boundary is rather wide with 250 km, but considering the lifetime of organized convective systems, usually exceeding 5 hours [37], we prefer to allow sufficient time for the development of these systems within the domain. In addition, we made a distinction based on the surface altitude, whereby we divided the data into areas below 400 m height and areas above that height. We term these areas the ‘low’ and ‘high’ altitude areas in the paper. A division height of 400 m was chosen to arrive at reasonably sized areas (figure 1). Also, a number of sub-areas (e.g. the Benelux and western part of France) are considered (see electronic supplementary material for plots of different analysis areas). The spatially and temporally pooled precipitation data was ordered, and we computed the ‘pooled fraction of exceedance’ (PFOE). This statistic is practically equivalent to the commonly used percentiles (PCTL)—with PFOE = 1 – PCTL/100, so e.g. the 99th percentile corresponds to a PFOE of 0.01 [31]. We mostly analysed the change in the distribution derived from all hours, but also used the wet hour conditioned distributions to investigate the influence of changes in the frequency of rain hours on extreme statistics [24].

For the climate change response in hourly extremes, derived from comparing the future simulation with the present-day simulation, changes are normalized by representative dew point temperature changes. These dew point temperature changes are derived from the dew point temperature data which is paired to the rainfall occurring 4 h later (as also used in the apparent scaling analysis). We used two measures: (i) ΔTd_wet_, the change in dew point temperature for wet events exceeding the threshold of 0.1 mm and (ii) ΔTd_P99_, the change in dew point temperature for rainfall events exceeding the 99th percentile of rainfall. The latter is more representative of the dew point temperature change of extreme rain events and is therefore used as the reference with which to scale the precipitation changes. To estimate uncertainty levels, we used a bootstrapping procedure (with replacement), sampling data blocks of 6 h and full fields (the same time blocks are used for both present-day and future periods). Plotted are 5–95% uncertainty ranges estimated from 100 resamples (note this is computationally quite expensive).

## 3. Results

### (a) Present-day scaling

**Figure 2.**
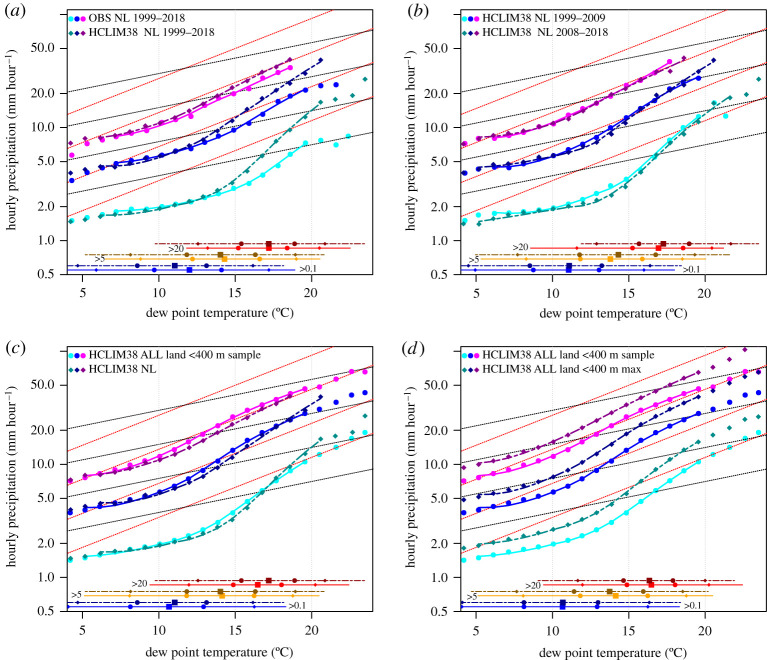
Scaling of hourly precipitation as a function of dew point temperature, showing three percentiles: 90 (cyan), 99 (blue) and 99.9th percentile (magenta). In each plot, two data sources are compared, labelled in the top left of the plot. The first data source (upper label) is shown in vivid/pure colours, and the second data source (lower label) is shown by the darker/muted colours. On the bottom, the dew point temperature ranges for precipitation exceeding 0.1, 5 and 20 mm is shown, with the 1 to 99 percentile range indicated by the line, and the 5,25,50,75,95th percentiles by the different markers. Straight stippled lines are CC (black) and 2CC (red) dependencies. (Online version in colour.)

We first evaluated the performance of the model HCLIM38 in a *perfect* boundary setting using the runs driven by reanalysis data (periods 1999–2009 for domain CXFPS and 2008–2018 for domain EUCP_NW, table 1). We compared the scaling derived from the model simulations to observations from approximately 30 hourly stations in the Netherlands (same as analysed in [7]). For the model, the closest grid point to the surface observation stations is chosen. The model faithfully reproduces the observed scaling relations for low to intermediate dew point temperatures up to 12°C (figure 2*a*). Yet, for higher dew point temperatures, the model overpredicts the rainfall intensities and simulates too strong dew point temperature dependencies. This is particularly true for the lower percentiles analysed, the 90 and 99th. The model results for the highest, 99.9th percentile are, however, in better agreement with the observations, only slightly overestimating the scaling rate. Using unconditional percentiles including dry hours, hardly affects these results; scaling rates are similar, consistent with results in [5] where similar discrepancies between model results and observations are found (see electronic supplementary material).

Besides dew point temperature dependencies, it is also revealing to look at the distribution of dew point temperatures for hourly rainfall exceeding a certain threshold (lines at the bottom in figure 2*a*). Hourly rain above 20 mm h^−1^ occurs at a median dew point of 17°C in both the model as well as the observations, and 90% of these events occur between 13°C and 21°C, with a slightly larger range in the model. Considering all rain hours, the modelled median dew point temperature is about one degree too low (11°C in the model versus 12°C in the observations). So, as expected, most of the extremes of hourly rainfall occur in a relatively high dew point temperature regime.

The scaling curves are quite insensitive to the time period selected, given an 11-year period and 32 grid points selected, representing in total approximately 400 years of hourly data. Results from the ERA-Interim driven simulations for the periods 1999–2009 and 2008–2018 are almost identical (figure 2*b*). Also, results are relatively insensitive to the region analysed. In figure 2*c*, we compare the scaling for the full domain, for the low-altitude land area only, and using values at the centre of the 5 × 5 grid boxes, Pr_sample_, with results for the selected Netherlands grid points, and find only marginal differences. Thus, scaling curves for this part of the world and the summer half-year are quite consistent and robust, both spatially and temporally.

Finally, to bridge the gap to the statistics used in the rest of the paper, we compared the scaling of the sample values, Pr_sample,_ with the maximum (over a grid of 5 by 5 points) values, Pr_max_. As expected, all percentiles of Pr_max_ are higher than Pr_sample_, but the dew point temperature dependencies are very similar (figure 2*d*).

### (b) Comparing present-day scaling with future scaling

**Figure 3.**
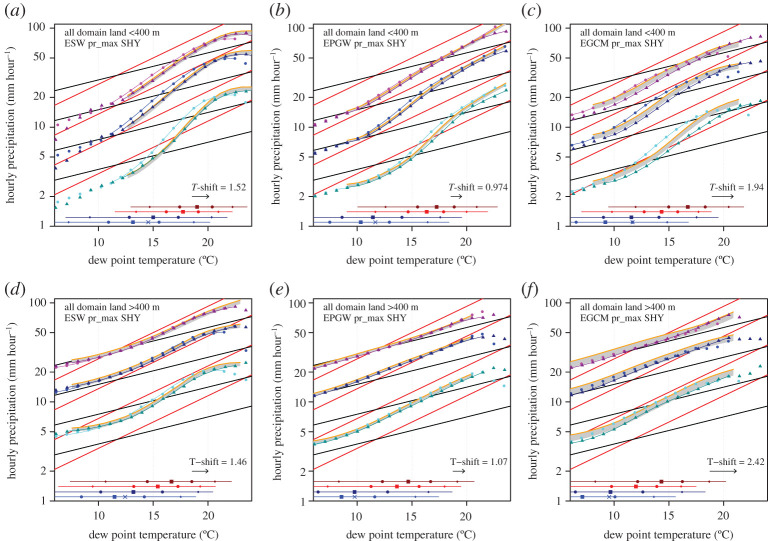
Scaling curves for present-day climate (vivid colours) compared to the future climate (muted colours), from left to right for E_SW_, E_PGW_ and E_GCM._ In addition, the projected range for the future scaling is shown, which is constructed by shifting present-day curves with a mean dew point temperature change based on heavy precipitation events, ΔTd_P99_, indicated by T-shift, and assuming CC and 2CC responses (2CC is given by the orange line). Colours indicate the 99.9 (magenta), 99 (blue) and 90th (cyan) percentile. Horizontal lines at bottom of graph are as figure 2, but only for 0.1 and 20 mm hour^−1^. (Online version in colour.)

Having established reasonably robust temperature scaling relations in the present-day climate, we now pose the question of how these relations are affected by climate change. A naïve assumption, partly rooted in the relative insensitivity in present-day climate as shown in figure 2*b*, is that the relations are unchanged; in the case of constant scaling rates (linear in log precipitation), this would imply that the scaling rates are a good predictor of climate change. Figure 3 shows that this is not a valid assumption for the low-altitude region. For all three experiments, major parts of the scaling curves in the future climate shift downward compared to present-day climate, in agreement with Zhang *et al*. [14]. This behaviour is most pronounced for the lower percentiles, but is not obtained for the highest dew point temperature range. The high-altitude region, surprisingly, shows a (much) better similarity between present-day and future scaling, in particular for the highest percentile where differences are very small for most of the dew point temperature range.

We note that the two experiments based on re-analysis data, E_SW_ and E_PGW_, display rather similar scaling for the present-day climate, despite the fact that E_SW_ is only based on a rather limited sample of 10 summer months. Yet, because these summer months were selected on the widespread occurrence of (heavy) precipitation, E_SW_ is biased towards moister conditions, with dew point temperatures approximately 2 degrees higher than E_PGW_ (horizontal lines at bottom of figure 3) and also higher percentiles of rain intensity (figure 5, values for 99th percentile).

To quantify the change of the scaling curves from present-day to future climate, we constructed future scaling curves by assuming a CC and a 2CC response, and shifted present-day scaling curves with a constant dew point temperature change (to the right) and enhanced rainfall amount according to the CC and 2CC rates [14,30]. For the dew point temperature shift, we used *Δ*Td_P99_, which is the dew point temperature change for events exceeding the 99th percentile, representing moderate to extreme events. The range given by these two projections is plotted as a grey band, with the 2CC rate-based projection denoted by the orange line, in figure 3.

**Figure 4.**
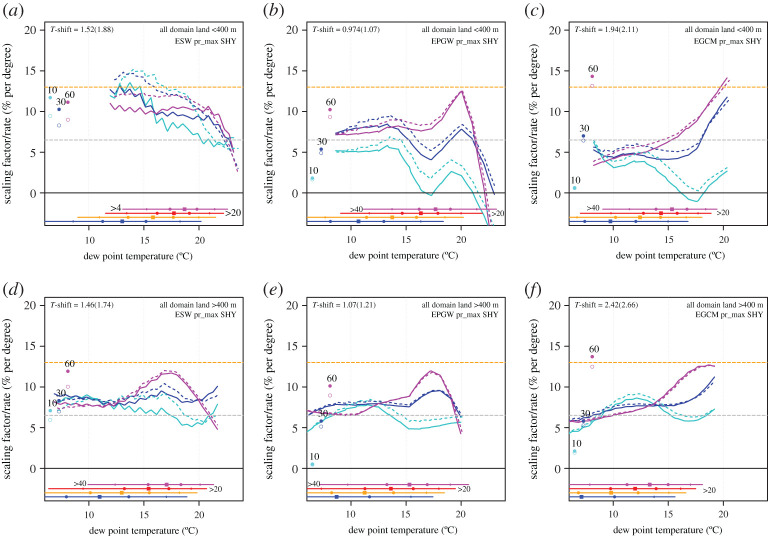
Scaling factors (% per degree dew point) derived from comparing the future scaling curves with the prediction given by shifting present-day curves by the change in dew point temperature (T-shift) representative for extremes ΔTd_P99_ (solid lines and dew point temperature given on *a*) and applying a scaling factor to the intensity. Dashed lines are derived using the dew point temperature change for all wet events ΔTd_wet_ (bracketed number for T-shift): (*a*,*b*,*c*) are derived for all land area below 400 m and (*d*,*e*,*f*) above 400 m. From left to right are the experiments: E_SW_, E_PGW_ and E_GCM_. The three dots at the left are the scaled climate change response from the pooled data at a fixed return level with an intensity of 10, 30 and 60 mm hour^−1^ in the present-day climate (solid dots scaled by ΔTd_P99_, open dots scaled by ΔTd_wet_*).* Colours indicate the 99.9 (magenta), 99 (blue) and 90th (cyan) percentile. (Online version in colour.)

Most of the future scaling curves fall within the band spanned by the CC-2CC prediction, with the exception of the 90^th^ percentile (in cyan) for E_PGW_ and in particular E_GCM_. However, this is arguably difficult to see in figure 3. Therefore, we further derived the dependency by comparing the future scaling curves with a prediction based on shifting the present-day scaling, and plotted the percentage change per degree, hereafter called the ‘scaling factor’, for each percentile and dew point in figure 4.

**Figure 5.**
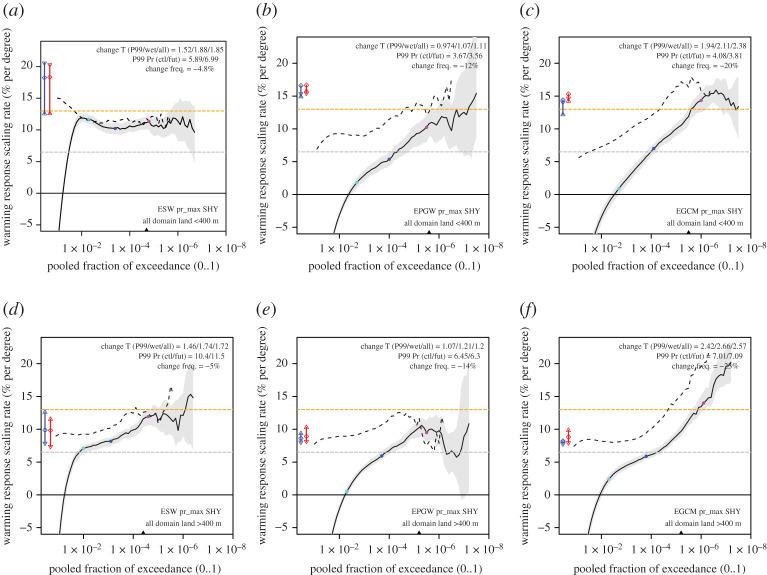
Climate change response (scaled by the dew point temperature change for ΔTd_P99_) as a function of the ‘pooled fraction of exceedance’ PFOE for low (*a*,*b*,*c*) and high (*d*,*e*,*f*) altitude land area. Solid lines are the unconditioned change rates, whereas the dashed lines are conditioned on the occurrence of rain (wet threshold is 0.1 mm hour^−1^). The change in the frequency of rain is shown at the top right in each panel. Grey and orange lines denote 1 and 2 times the CC rate, respectively. The blue (control) and red (future) lines and symbols on the left denote the scaling rates derived from the scaling diagram by fitting to the lower dew point temperature range (downward facing triangle), the full range (circle) and the upper dew point temperature range (upward facing triangle), respectively. The colour dots indicate the positions where rainfall amounts for the present-day simulation are 10 (cyan), 30 (blue) and 60 (magenta) mm hour^−1^, respectively, and the triangle at the bottom marks the PFOE belonging to the top 1000 rainfall hours. (Online version in colour.)

Analysing only the area below 400 m, the scaling factors of the simple SW experiment, E_SW_, reveal the most regular behaviour, usually with change factors ranging between 9 and 13% per degree, with the exception of the behaviour of the 90th percentile for dew point temperatures above 15°C (figure 4*a*). Thus, repeating the same events under warmer and moister conditions, with a relatively small change in temperature stratification, leads to a shift in precipitation distributions in agreement with 1.5–2 times the CC rate. Yet, the high-altitude area (greater than 400 m) shows a different behaviour, with intensities increasing at the CC rate (or slightly above) for most of the percentiles and dew point temperature range (figure 4*d*). However, the most extreme events as represented by the highest percentiles appear to have substantially higher change factors for dew points between 15 and 20°C, with scaling factors similar to the low-altitude area.

The more realistic experiments, E_PGW_ and E_GCM_, both show a different behaviour for the low-altitude area (<400 m) compared to E_SW_. Change factors are typically close to the CC rate for dew points up to 15°C and for all percentiles. E_PGW_ has somewhat higher scaling factors for the highest two percentiles, and E_GCM_ typically shows a scaling factor of 5% per°C, somewhat below the CC rate, for dew points below 15°C. Contrastingly, the response patterns change dramatically for dew point temperatures above 15°C . The lowest percentile appears to hardly respond to warming, with scaling factors close to zero or marginally positive at up to 3% per°C. Yet, the highest percentile, the 99.9th, shows a sensitivity increasing with dew point temperature, peaking at close to the 2CC rate at a dew point of 20°C.

One feature of the low-altitude area scaling factors in the two climatic perturbation-based experiments, E_SW_ and E_PGW_, is the decreasing scaling rate for dew point temperatures beyond 20°C. In E_SW_, a gradual drop in sensitivity is seen for all percentiles, whereas in E_PGW_, it is a more rapid transition. While this may seem to conflict with a simple scaling argument, it may actually be qualitatively consistent with the scaling curves in figure 3: above a dew point of 20°C, the scaling curves do show a decrease in the sensitivity of hourly precipitation extremes to dew point temperature. We think this is likely a physical effect as the flattening of the scaling curves was also obtained in observed precipitation from Hong Kong, where there is ample data in the high dew point temperature regime [7]. Finally, we note that E_GCM_ does not show a decreased sensitivity beyond a dew point of 20°C, and this may well be related to the fact that E_GCM_ is rather cold (and dry) and therefore has too little data in this high dew point temperature regime to derive the scaling factors (see also the dew point temperature ranges for each percentile at the bottom of figure 3).

The scaling factors for the high-altitude area (greater than 400 m) reveal a surprisingly similar behaviour for all three experiments (figure 4 *d*,*e*,*f*). They all show approximately CC behaviour up to a dew point of 15°C for all percentiles and higher scaling factors for the highest percentile, peaking at 12–13% per degree at a dew point of 18–20°C. Beyond that dew point temperature scaling factor appears to decrease, most visible for E_SW_ and least visible for E_GCM_ (see also electronic supplementary material for sensitivity to interpolation).

Admittedly, the analysis here is somewhat sensitive to the technical details of the scaling analyses (bin width and smoothing from the interpolation; see electronic supplementary material) and the representative dew point temperature change used to derive the scaling factors. To show the sensitivity to the latter, we also computed the scaling factors based on the dew point temperature change derived for all wet hours, and results are shown by the dashed lines in figure 4. This generally leads to somewhat higher scaling factors, in some cases up to 2% per degree, but does not change the general conclusions.

In summary, it is clear that apparent scaling curves are reasonably robust, but nevertheless do depend on the climatic state and are therefore altered by anthropogenic climate change. The predictive value of *local* (i.e. at a certain dew point) scaling rates appears limited, with the potential exception of the decreasing sensitivity beyond 22°C dew point temperature which appears to be reflected in the scaling factors. Yet, by comparing present-day with future scaling curves, there *is* evidence that super-CC responses, even up to the 2CC rate, with warming are possible. In the next section, we will continue by looking at the full (not conditioned on dew point temperature) climate change response in hourly extremes from the pooled data.

### (c) Linking apparent scaling to the climate change response

We start by analysing the changes in hourly precipitation from the pooled data of pr_max_ over land using the low-altitude area selection (figure 5*a*,*b*,*c*). Here, we focus on unconditional statistics that include the dry periods (solid lines), and compare those to wet-conditioned statistics (dashed line). Using the unconditioned percentiles avoids a positive bias in the response in case of a decrease in the frequency of wet hours, which is typical for the summer response in the area considered [24] and which can be seen by comparing the unconditional response to the conditional response (compare dashed with solid line in figure 5).

The simple experiment, E_SW,_ reveals a very consistent response of 11% per degree, not dependent on return level (value of the pooled fraction of exceedance, PFOE). Here, we normalized the change in precipitation extremes with dew point temperature change representative for extremes above the 99th percentile (ΔTd_P99_, typically exceeding 4–7 mm per hour, numbers given in the plot). The flat response in figure 5*a* appears consistent with the rather uniform scaling factors derived from the scaling analysis, and the values correspond quite well (compare in figure 4 the dots on the left, which correspond with the coloured dots on the solid lines in figure 5, with the lines representing the scaling factors).

The more realistic experiments, E_PGW_ and E_GCM_ show a drastically different climate change response in pr_max_. Both clearly show much lower climate change sensitivities, below the CC rate, for moderate extreme events with return levels that correspond to 30 mm h^−1^ in the control climate (the blue dot on the curve in figure 5). However, for the most extreme events, the rate of intensification increases, with responses up to the 2CC rate for the most extreme events. This tail behaviour is close to the rate that is predicted by the scaling analysis in figure 4 (red and blue symbols at the left of the plot in figure 5). Obviously, uncertainty levels are considerable for these extremes, but even taking this into account the response of the most extreme hourly precipitation intensities is clearly above the CC rate.

Taking only the wet hours into account the response is higher, which is unsurprising as the frequency of wet hours decreases in the future projections, by only a small amount in E_SW_ (−5%), but more strongly in E_PGW_ (−12%) and E_GCM_ (−20%). Clearly, even in E_SW,_ where no changes in circulation are imposed, decreasing frequencies of rain are obtained, pointing at a reduction in the frequency of light rainfall events. Yet, for wet hours exceeding 10 mm h^−1^ the changes are quite stable, and the distribution of the hourly rainfall appears to increase by the same factor. In E_PGW_ and E_GCM_, a much bigger influence of the decrease in the frequency of wet hours is seen. The wet hour conditioned changes are much larger and range between the CC rate (for moderate events) to the 2CC rate at the extreme tail. For the most extreme wet hours, the conditional statistics appear to converge to the unconditional case, which is expected behaviour considering the exponentially decaying tail of the rainfall distribution [5,38].

The climate response for the high-altitude area is less conclusive. The tendency for increases in the climate response scaling rate is now also visible for the simple E_SW_ and is shown in all experiments. Yet, the behaviour of the far tail is less univocal, with a sharp increase in the rate in the GCM driven experiment, but less pronounced behaviour in the other two experiments.

Considering the apparent scaling rates derived from fitting to the scaling curve of the 99.9th percentile (coloured bars at the left of figure 5), it appears that for the low-altitude areas the tail behaviour—a dependency close to 2CC—is predicted rather well for E_PGW_ and E_GCM_, but overestimated for the more simple E_SW_. The latter overestimation is partly due to the fact that the apparent scaling rate is biased because the experiment has been set up around the most wet (and therefore humid) months from a 10-year period [31]. But even when taking the more realistic apparent scaling rates from E_PGW_ (also reanalysis-based but for a longer period), the climate change response is still lower. We hypothesize that this is related to the stabilization of the atmosphere. So, why do E_PGW_ and E_GCM_ converge to higher responses in the tail? This could be related to the decrease in relative humidity in both experiments, which implies that for the same dew point temperature the instability will increase, with a lower relative humidity implying a higher surface temperature. The compensation of upper air warming by enhanced surface warming has been noted in previous studies [4,39]. While the atmospheric stability (in terms of the dry lapse rate) increases on average in the future climate, the atmospheric conditions for extreme events may deviate from this and be more unstable than the mean stabilization suggests; see also the larger changes in CAPE values in the tail of the distribution in the supplement of [40].

**Figure 6.**
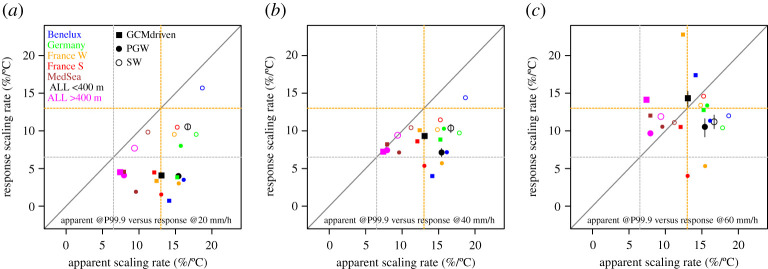
Climate response scaling rate compared to apparent scaling rate for intensities of 20 (*a*), 40 (*b*) and 60 (*c*) mm hour^−1^ in the present-day simulation for a number of sub-regions and the three experiments: E_SW_, E_PGW_ and E_GCM_. Regions are low-altitude land areas only (see electronic supplementary material for plots of these sub-regions), and ‘ALL’ is the analysis area used in the main text (figure 1). (Online version in colour.)

Finally, we investigated the climate response in a number of different sub-regions and compared these to the apparent scaling rates. We compared the climate response rate for three different intensity classes, based on the pooled fraction of exceedance belonging to intensities of 20, 40 and 60 mm h^−1^ in the present-day simulation. Clearly, for the lowest intensity class (figure 6*a*), the apparent scaling rates overpredict the climate response by a considerable amount. We note that this is the least so for E_SW_ where there is already a fair agreement between apparent scaling rates and climate change response rates (open symbols in figure 6).

For more extreme hourly precipitation intensities, the climate response rate steadily increases, and on average, they appear to correspond to the apparent scaling rates for the most extreme hourly intensities, exceeding 60 mm h^−1^ in the present-day simulation. Yet, on an individual level, there is no such correspondence between the apparent scaling rates and the climate change response rates for the different sub-regions. Interestingly, for some sub-regions, results are very different, for instance showing the highest climate response in E_GCM_ for Western France, but the same region shows (almost) the lowest climate response in E_PGW_. Obviously, even though we have pooled relatively large areas, the signal-to-noise ratio at the sub-region scale is too low for these extremes to draw firmer conclusions.

## 4. Discussion

We used the surface dew point temperature taken at the same location a few hours before the rain event in our analysis. In the concept of a simple updraft model of a convective plume, the near-surface dew point temperature is a good estimate for the cloud base temperature (and humidity, since the cloud base is at saturation). So, we think that the surface dew point temperature is a good proxy for the moisture entering a convective cloud at cloud base. One may question, however, whether this is the most appropriate scaling variable as the humidity source of a convective shower may originate more from the mid-levels, instead of from the atmospheric boundary layer, or may be advected into the cloud from larger distances. For instance, in a simple budget analysis of a convective plume, it was found that half of the moisture enters the plume above cloud base [4], and the moisture convergence also depends on the profile of the updraft [41]. Yet, we also notice that taking the preceding surface dew point temperature appears to result in the most consistent apparent scaling behaviour, both over the (dew point) temperature range, between percentiles and also comparing different regions [5,7,12,13,25].

With respect to the climate change response, the appropriate scaling variable is also debated in the literature, and local, regional and also globally averaged (dew point) temperatures are commonly used. Taking the mean surface temperature change, which is approximately two times larger than our measure of dew point temperature change in E_PGW_ and E_GCM_, would result in lower climatic scaling, more consistent with the CC rate. To strengthen our case, we notice the consistency between the scaling factors (derived from shifting the distributions conditioned on dew point temperature) and the climate change scaling rate. This is most clearly visible for experiment E_SW_ (figure 4*a*), which is only weakly influenced by changes in the frequency of wet events and repeats present-day weather in warmer conditions leading to well defined, rather uniform response of hourly extremes. This is a non-trivial result, as taking a different value for the dew point temperature change, for instance derived for all rain hours, leads to inconsistency with scaling factors (dashed lines) being systematically higher than the climate change scaling rate (open symbols).

What is also striking is the relatively low values we obtained for the representative dew point temperature change. In the full GCM-driven experiment, for the end of the century compared to the present using a high-end emission scenario, it is only around 2°C; the corresponding global temperature change in the GCM is more than 3.5°C. Part of the lagging of the dew point temperature change can be attributed to decreases in relative humidity, but it is also conceivable that changes in the timing of convection during the day [42] or the seasonality [43] play a role here.

Considering the climate change experiments, E_SW_ only models the thermo-dynamical response directly related to increasing moisture (including cloud feedbacks on for instance atmospheric stability). For this case, we obtain reasonably consistent precipitation sensitivities for the low-altitude area (less than 400 m), which are rather insensitive to extremity, percentile or dew point temperature. Most of the sensitivities are near to 11% per°C, about 2% per°C below the 2CC rate. Partly this lower sensitivity could be due to the stabilization of the atmosphere, which is not as strong as for a fully GCM-driven simulation, but still present [31]. Whereas E_SW_ reveals the most regular response patterns of hourly extremes, it is also the least realistic experiment representing climate change since it neglects a number of important changes, such as in large-scale circulation, in relative humidity, and a more pronounced stabilization of the atmosphere.

The other two experiments, E_PGW_ and E_GCM_, are more realistic since they include changes in relative humidity, circulation and lapse rate. These changes clearly affect the frequency of occurrence of rain; domain-averaged decreases in rainfall frequency of 20% are obtained for the summer half-year and an even higher reduction in summer only. On the one hand, decreases in relative humidity may result in higher levels of convective inhibition, and higher cloud base heights, which decreases the frequency of rain. On the other hand, decreases in relative humidity are also associated with higher potential instability due to higher surface temperatures, which increases the potential for heavy rain. Moreover, decreases in relative humidity may affect cloud processes, for example, promoting the formation of cold pools [44] that play an important role in boundary layer moisture convergence, but at the same time also promoting cloud dissipation [45].

For dew point temperatures above 15°C, we consistently obtained higher scaling factors for the highest rainfall percentiles (figure 4), with rates peaking close to 2CC between 15 and 20°C (except for the E_SW_, low-altitude area, which remains rather flat). It appears that due to dynamical feedbacks caused by latent heating (and evaporation of rain causing downdrafts and cold pools) the strongest showers intensify more with warming at the expense of small showers, and there is no unique change rate for all showers. This finding confirms results from very high-resolution (at 200 m) simulations of convective showers at different warming levels [46].

Results also reveal that for dew point temperatures exceeding 20–22°C, sensitivities of rain extremes to dew point temperature may decrease again. This is obtained for the apparent scaling rates (figure 3), but also for the climate scaling factors (figure 4). We do not know the reason for the levelling off behaviour, but we speculate that limitations in the microphysical processes [47], and the area growth of rain systems [48] potentially set by the height of the tropopause may play a role. We remind here that a very distinct levelling off for dew points above 23°C has been obtained in observations from Hong Kong [7]. Besides being related to physical processes, we note that the S-shape of the apparent scaling curve may also be (partly) explained by statistical effects, for instance due to the mixing of rain types [18,19].

While our model is convection-permitting and explicitly resolves the largest convective motions, and improves on many aspects, like intensity and diurnal cycle [32], of convective rain, it does not fully resolve all convective processes. It is still an open question how even smaller scale processes, like cloud entrainment and boundary layer cold pool dynamics, and cloud microphysics, could affect the sensitivity of convective rain to warming [45].

## 5. Conclusion

We performed three sets of experiments with a convection-permitting climate model to study the climate change response of hourly precipitation extremes in relation to apparent scaling on dew point temperature derived from day-to-day variability. As in previous studies, the so-called apparent scaling rate usually exceeds the CC relation (6–7% per degree) and typically takes a value close to two times the CC rate (2CC). Climate change scaling rates (when scaled with the dew point temperature change representative for extremes) can also exceed the CC rate. For the simplest experiment, where the atmosphere is warmed assuming unchanged relative humidity (labelled E_SW_), typical climate change response rates are 10–12% per°C, approximately 2–4% per°C lower than the apparent scaling rates, and the response is not dependent on return level. For the more realistic experiments—E_PGW_, a full pseudo-global warming approach, and E_GCM_, a GCM-driven experiment—the climate change response strongly depends on the return level. Moderate extremes usually increase at rates close to the CC rate (or even below). Yet, the most extreme hourly rainfalls, with typical intensities beyond 50 mm h^−1^, increase at a rate approaching 2CC, which is in reasonable agreement with the apparent scaling rates and results from another recent CPRCM study [49]. This has important implications for the impacts of flash flooding events and hence climate adaptation planning.

The precipitation apparent scaling diagram is affected by climate change, and usually percentiles, and in particular, the lower ones shift downward to lower intensities. So, apparent scaling is not invariant to climate change and local gauge-level scaling rates inferred from day-to-day variability are in general not straightforward predictors of climate change related increases of precipitation extremes. The shift in the scaling diagram from the present day to the future climate is usually compatible with the CC rate, consistent with previous studies [14,30]. However, changes in the scaling diagram also display super-CC behaviour. For the simple uniform-warming experiment E_SW_, the change in the scaling diagram is best explained by a 10–12% per°C sensitivity, which is in agreement with the overall change in intensity of precipitation extremes. The E_PGW_ and E_GCM_ experiments take more realistic changes in large-scale patterns of circulation change into account, which in both experiments is based on the global climate model ECEARTH. In general, results from both experiments are comparable, suggesting that the omission of changes to day-to-day variations in large-scale circulation in E_PGW_ is not a major controlling factor. Both experiments reveal changes to the scaling diagram more consistent with the CC rate for low dew point temperatures. Nevertheless, in a range of dew point temperatures between 15 and 20°C, the most extreme percentiles do reflect super-CC behaviour, of up to 10–13% per°C. This is also the dew point range where most of the strong extremes occur, so this super-CC behaviour is strongly reflected in the response of the extreme tail of the distribution.

In conclusion, we have shown evidence of super-CC behaviour in changes to hourly extreme precipitation intensities in three sets of climate model simulations. While temperature scaling is definitely not a one-size-fits-all solution and should be interpreted with care, we have shown here that it does provide useful information on how precipitation extremes may respond to future warming. Our results indicate that in high-humidity regimes extreme hourly precipitation intensities could be more sensitive to warming than suggested by the increase in atmospheric moisture alone, with the most extreme intensities showing the strongest increases. This also poses additional questions with regard to the physical processes involved.

## Data accessibility

Re-gridded modelling data can be obtained by contacting geert.lenderink@knmi.nl. Observational data for the Netherland from https://www.knmi.nl/nederland-nu/klimatologie/uurgegevens.
